# Increased expression of long non-coding RNA FIRRE promotes hepatocellular carcinoma by HuR-CyclinD1 axis signaling

**DOI:** 10.1016/j.jbc.2024.107247

**Published:** 2024-03-29

**Authors:** Yuki Haga, Debojyoty Bandyopadhyay, Mousumi Khatun, Ellen Tran, Robert Steele, Sumona Banerjee, Ranjit Ray, Mustafa Nazzal, Ratna B. Ray

**Affiliations:** 1 Department of Internal Medicine, Saint Louis University, St. Louis, Missouri, USA; 2 Department of Pathology, Saint Louis University, St. Louis, Missouri, USA; 3 Department of Surgery, Saint Louis University, St. Louis, Missouri, USA

**Keywords:** lncRNA FIRRE, hepatocellular carcinoma, HuR, cell growth, tumor progression, biomarker

## Abstract

There is a critical need to understand the disease processes and identify improved therapeutic strategies for hepatocellular carcinoma (HCC). The long noncoding RNAs (lncRNAs) display diverse effects on biological regulations. The aim of this study was to identify a lncRNA as a potential biomarker of HCC and investigate the mechanisms by which the lncRNA promotes HCC progression using human cell lines and *in vivo*. Using RNA-Seq analysis, we found that lncRNA FIRRE was significantly upregulated in hepatitis C virus (HCV) associated liver tissue and identified that lncRNA FIRRE is significantly upregulated in HCV-associated HCC compared to adjacent non-tumor liver tissue. Further, we observed that FIRRE is significantly upregulated in HCC specimens with other etiologies, suggesting this lncRNA has the potential to serve as an additional biomarker for HCC. Overexpression of FIRRE in hepatocytes induced cell proliferation, colony formation, and xenograft tumor formation as compared to vector-transfected control cells. Using RNA pull-down proteomics, we identified HuR as an interacting partner of FIRRE. We further showed that the FIRRE-HuR axis regulates cyclin D1 expression. Our mechanistic investigation uncovered that FIRRE is associated with an RNA-binding protein HuR for enhancing hepatocyte growth. Together, these findings provide molecular insights into the role of FIRRE in HCC progression.

Hepatocellular carcinoma (HCC) is the dominant type of liver cancer (70–80%) and accounts for >75% of the cases worldwide (1). HCC is a deadly disease with limited treatment options. Fortunately, modern medicine has achieved to develop antiviral agents that transformed standard therapy against hepatitis B virus (HBV) or hepatitis C virus (HCV), the major etiologies for HCC, and associated mortalities. Unfortunately, the therapies fail to completely eradicate liver cancer risk, especially in HCV-cured patients with advanced liver disease (2, 3, 4, 5). Further, Hensel *et al.* (6) reported that antiviral treatment of HCV-infected individuals does not repair T-cell function, leading to end-stage liver disease. Recent studies also suggested that individuals who received direct-acting antiviral (DAA) treatment for HCV infection remain at an elevated HCC risk (7). These data suggest that chronic HCV infection causes an accumulation of irreversible liver damage. In fact, HCV proteins modulate several host molecules, including noncoding RNAs, leading to neoplasia (8). Many studies suggested that the accumulation of liver damage during chronic HCV infection is caused by a complex interaction of direct and indirect mechanisms (8), forcing the liver towards a tipping point of no return from HCC development in some cases. Cirrhosis is an important factor in HCC development since most HCCs occur in cirrhotic livers. Non-HCV risk factors, such as HBV infection, alcoholic liver disease, and non-alcoholic fatty liver disease, can perpetuate liver inflammation, tissue injury, and epithelial regeneration, leading to HCC development (4, 9). HCC is exacerbated by the problem of late diagnosis, and many HCC patients are asymptomatic at early stages but have severe consequences with poor (<20%) overall 5-year survival rate (10).

Transcriptome studies and genome tiling arrays show that >90% of the human genome transcribes noncoding RNAs, that is, RNAs with no protein-coding potential (11, 12). Long noncoding RNAs (lncRNAs) are distinguished from other noncoding RNAs (such as microRNA, circular RNA, and Piwi-interacting RNAs) as having >200 nucleotides in length. LncRNAs have recently gained widespread attention as a new layer of regulation in biological processes (13, 14, 15). In recent years, lncRNAs have emerged as a promising class of targetable molecules with immense potential in cancer treatment (16, 17, 18, 19).

In this study, we report that lncRNA FIRRE is upregulated in HCC specimens compared to normal liver tissues. Overexpression of FIRRE promotes tumor growth in HCC xenograft mice. Increased expression of FIRRE was noted in HCC tissues suggesting the potential of FIRRE as an oncogene and may serve as an additional biomarker for HCC. Further, mechanistic investigations into the function of FIRRE in HCC suggested that it interacts with the RNA binding protein HuR, as evident by RNA-IP-proteomics analysis. FIRRE-HuR axis modulates the downstream molecule, cyclin D1, an important regulator of the cell cycle progression pathway of cancer.

## Results

### Overexpression of lncRNA FIRRE in HCV-associated HCC

**Figure 1:**
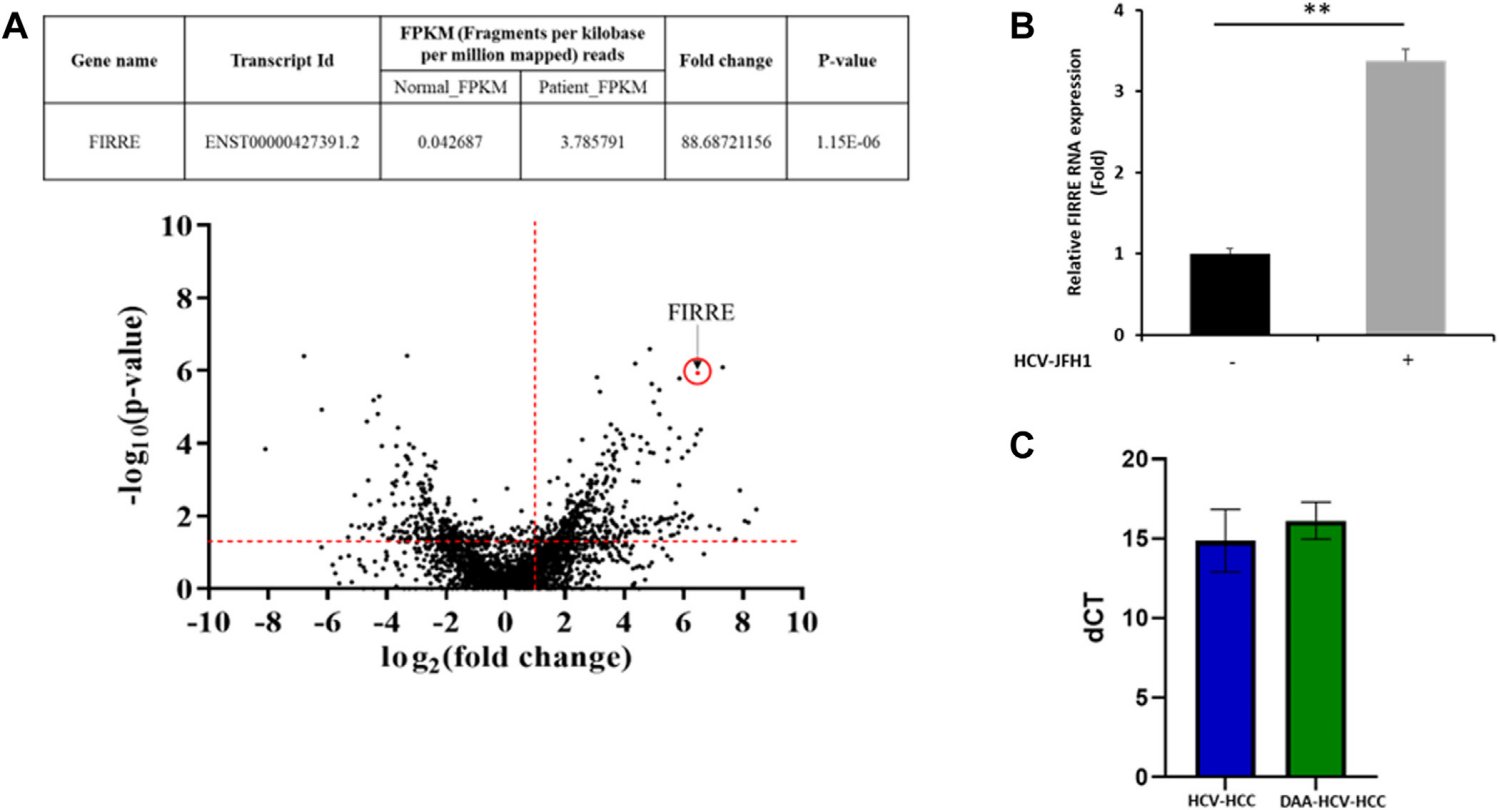
**FIRRE is upregulated in HCV-associated liver cancer samples.***A*, the fragments per kilobase of transcript per million mapped reads (FPKM) counts of FIRRE from non-tumor *versus* tumor samples from RNA-seq (*top panel*). A volcano plot of the RNA-seq data is shown (*bottom panel*). FIRRE is upregulated in HCC samples compared to control (adjacent non-tumor tissue). The x-axis is the log2-fold change value, and y-axis is the log10-*p*-value showing statistical significance. The *horizontal dashed line* marks *p* = 0.05 (−log_10_(0.05) = 1.3), and the *vertical dashed line* indicates the fold change at 2 (log_2_(2) = 1). The absolute 2-fold change and *p*-value 0.05 are used as the threshold cut-off. *B*, Huh7 cells were infected with HCV-JFH1 (moi = 1.0). Total RNA was isolated from mock-treated or virus infected cells. FIRRE and 18S rRNA were measured by qRT-PCR. Data were analyzed by Student's *t* test, and relative gene expression is shown with mean ± SD (∗∗*p* < 0.01), n = 3 biological replicates and 2 technical replicates. *C*, RNA isolated from HCV-HCC (n = 3), and DAA-treated HCV-cured HCC (n = 3) resected specimens were used to examine FIRRE expression by qRT-PCR with 3 technical replicates. 18S rRNA was used as an internal control. Data are presented as dCT, and small bars indicate SD.

LncRNAs have been implicated in a diverse array of human cancers. To understand the global gene expression pattern, we performed RNA-Seq analysis using RNA from HCV-associated HCC, and corresponding adjacent non-tumor tissues. We found that lncRNA FIRRE is significantly upregulated (∼88 fold) in HCV-associated liver tissue compared to adjacent non-tumor tissue (Fig. 1*A*-top panel). A volcano plot of -log_10_(*p* values) *versus* log_2_(fold changes) of FPKM reads was constructed to display the transcriptomics data from quantitative analyses, including FIRRE (Fig. 1*A*-bottom panel). We next examined the status of FIRRE in Huh7 cells infected with HCV JFH1 (multiplicity of infection = 1.0) and observed higher expression (>3.5 fold) in virus-infected cells as compared to mock-infected cells (Fig. 1*B*). Next, we tested whether HCV needs to be present for the upregulation of FIRRE. We compared the FIRRE expression between HCV-HCC and DAA-treated HCV-cured HCC specimens and observed similar expression level in both cases (Fig. 1*C*). We previously observed high c-Kit expression and virus replication in HCV-infected primary human hepatocytes until day 3 to 4, although, HCV RNA was almost undetectable on day 42, whereas c-Kit expression remained high (20). Therefore, our results suggested that once the FIRRE gene is upregulated following HCV infection, the expression is maintained at a high level even after the HCV cure following treatment.

### Ectopic expression of lncRNA FIRRE accelerates hepatocyte proliferation

**Figure 2:**
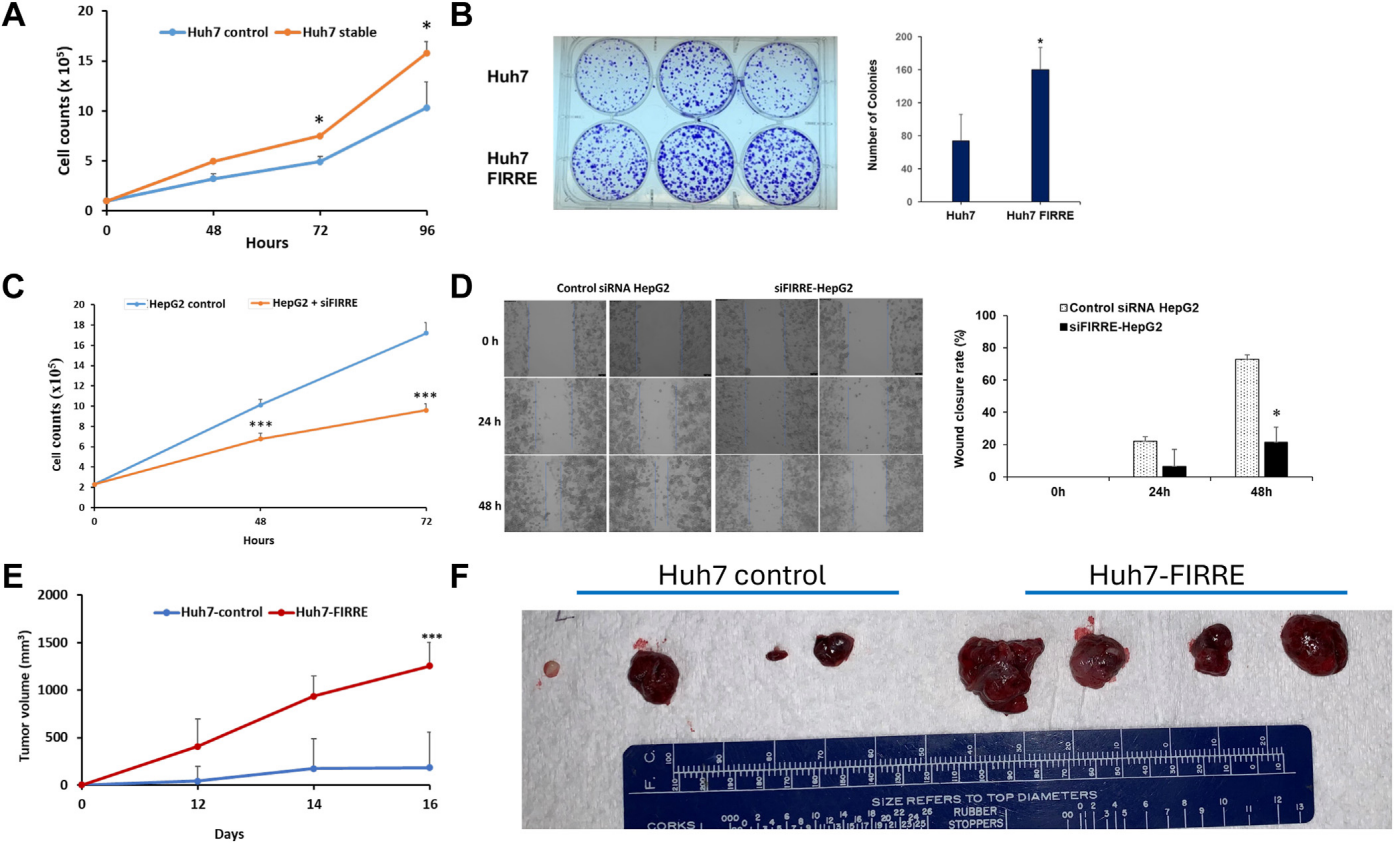
**Overexpression of FIRRE enhances hepatocyte growth.***A*, control (empty vector) or stably transfected FIRRE overexpressing Huh7 cells were analyzed for cell proliferation using the Trypan blue exclusion test at the indicated time points. N = 3 biological replicates and two technical replicates. Live cell numbers are presented, and small bars indicate SD. *B*, control or FIRRE overexpressing Huh7 cells (3 × 10^2^) were seeded and allowed to form colonies. The colonies were stained with crystal violet and counted after 2 weeks. Representative images of colonies in vector control and FIRRE overexpressing cells are presented. The *right panel* shows colony numbers obtained from biological triplicates, and represented as mean ± SD. N = 2 biological replicates and three technical replicates. *C*, HepG2 cells were transfected with control or FIRRE siRNA (20 nM), and cell proliferation was analyzed at indicated time points (n = 3 biological replicates, and two technical replicates), and data representsas mean ± SD. *D*, HepG2 cells transfected with control or FIRRE siRNA (20 nM) were seeded at confluency. Representative images of wound healing (at 0, 24 and 48 h after scratch) are shown (*left panel*) (magnification: 20× and black scale bar 75 μm). The *right panel* shows wound closure obtained from biological triplicates, and represented as mean ± SD. N = 3 biological replicates, and two technical replicates. *E* and *F*, Huh7 control or FIRRE overexpressing Huh7 cells (Huh7-FIRRE) were injected into the flank of nude mice (n = 4). Tumor volume was measured at indicated times and presented as a mean ± SD. Representative images of tumors from control and experimental groups are shown. Data information: Data were analyzed by Student’s *t* test. Small bars indicate SD (∗*p* < 0.05; ∗∗*p*< 0.01; ∗∗∗*p* < 0.001).

We observed differential expression of FIRRE in six HCC cell lines when compared to normal hepatocytes (Fig. S1). Based on the expression level, we generated stable expression of FIRRE in Huh7 cells with low basal FIRRE expression levels. By performing Trypan blue exclusion and colony formation assays, we found that Huh7 cells with stable FIRRE expression significantly induced cell proliferation and colony formation, compared with that of parallel stable cells containing the empty vector control (Fig. 2, *A* and *B*). On the other hand, the knockdown of endogenous FIRRE expression reduced the proliferative capacity of HepG2 cells (Fig. 2*C*). We chose the HepG2 hepatoblastoma cell line for the FIRRE knockdown assay since the expression of this lncRNA is much higher in these cells as compared to other HCC cells. We further determined the ability of FIRRE in HepG2 cells for modulation in migration using an *in vitro* wound-healing motility assay. FIRRE knocked-down HepG2 cells, seeded in a culture dish, were scratched with a thin disposable tip to generate a wound in the cell monolayer, and the cells were incubated for 48 h and photographed. FIRRE knocked-down HepG2 cells were less proficient in closing an artificial wound created over the confluent monolayer than control-siRNA transfected cells (Fig. 2*D*, left panel). The quantification of wound closure was presented (Fig. 2*D*, right panel).

The growth-enhancing effect of FIRRE was also verified by performing *in vivo* tumor growth assays. Xenograft tumors grown from cells overexpressing FIRRE had larger mean volumes and formed more rapidly, than control cells (Fig. 2, *E* and *F*). Huh7 cells overexpressing FIRRE displayed accelerated tumor growth in mice with an average tumor volume of >1000 mm^3^ in 14 days and continued to increase in size compared to control lncRNA-transfected Huh7 cells (>300 mm^3^) in the same period. Since we observed faster tumor growth in FIRRE overexpressing Huh7 cells, we examined whether VEGF (angiogenesis marker) or epithelial–mesenchymal transition marker (Oct4) is altered in these tumors. Our results show an increased expression of VEGF and Oct4 in Huh7-FIRRE cell implanted tumors as compared to control Huh7 cell implanted tumors (Fig. S2). We also observed higher expression of Oct4 in Huh7-FIRRE cells as compared to control cells (Fig. S3). Together these results suggested that FIRRE expression can promote HCC cell proliferation both *in vitro* and *in vivo*.

### LncRNA FIRRE interacts with HuR

**Figure 3:**
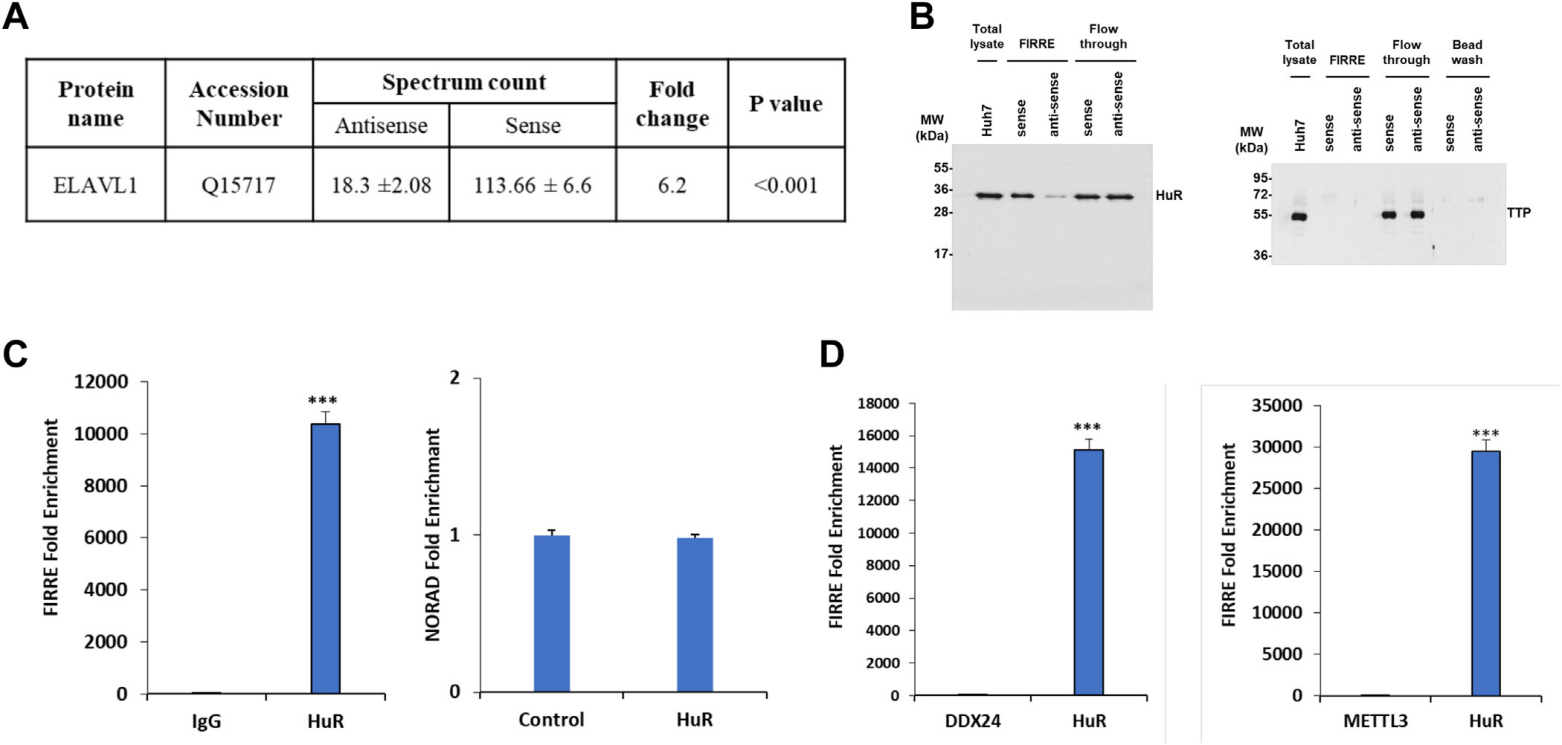
**FIRRE interacts with ELAVL1/HuR protein.***A*, spectrum counts of antisense or sense FIRRE RNA for ELAVL1 from MS. *B*, Western blot analysis was performed from biotinylated FIRRE sense and anti-sense RNA pull-down from Huh7 cell lysates using a specific antibody and the presence of HuR is indicated. The blot was reprobed with an antibody to TTP (an unrelated RNA binding protein) for specificity. *C*, cell lysates from FIRRE plasmid DNA-transfected cells were immunoprecipitated using isotype control (IgG) or HuR antibody. RNA was isolated from the immunoprecipitates and the relative enrichment of FIRRE (*left panel*) or NORAD (*right panel*) was analyzed by qRT–PCR. *D*, RNA immunoprecipitation assay was performed with unrelated antibody DDX24, METTL3 or HuR as a positive control and FIRRE expression was detected by qRT-PCR. Data information: n = 3 biological replicates and three technical replicates. Data were analyzed by Student's *t* test. Small bar indicates SD (∗∗∗*p* < 0.001).

Little is known about FIRRE-interacting molecules in HCC. To identify FIRRE interacting partners, we performed an RNA pull-down analysis followed by mass-spectrometry using sense or anti-sense strands of biotin-labeled FIRRE RNA. The mass spectrometry analysis identified 4495 proteins, and we identified ELAVL1/Human antigen R (HuR) as one of the interacting partners with a high spectral count in the FIRRE sense strand as compared to the anti-sense strand (6.2-fold) (Fig. 3*A*). HuR is a well-documented RNA-binding protein and binds to adenine-uridine-rich elements (AREs) containing mRNA to protect from rapid degradation in the cytoplasm or inhibit translation interaction with other ARE-binding proteins. HuR is overexpressed in HCC and regulates cell proliferation and survival (21, 22). TCGA data also indicated higher expression of HuR in liver cancer compared to normal tissue.

Next, we verified the RNA-protein interaction by using biotinylated sense or antisense FIRRE RNA pulled down from Huh7 cell lysates, followed by Western blot analysis with HuR antibody, and observed the presence of HuR in RNA-protein complex (Fig. 3*B*-left panel). To further verify the specificity of FIRRE-HuR interaction, we performed a Western blot with tristetraprolin (TTP) antibody. TTP is an RNA-binding protein and binds to AREs within the 3′ untranslated region (3′UTR) causing destabilization of mRNAs. However, we did not observe the presence of unrelated RNA binding protein TTP in FIRRE pull-down lysates, indicating the specificity of FIRRE-HuR binding (Fig. 3*B*-right panel).

In a reciprocal experiment, we verified the interaction between FIRRE and HuR using RNA immunoprecipitation (RIP) analysis using HuR antibody, followed by qRT-PCR. FIRRE RNA was significantly enriched in Huh7 immunoprecipitates (Fig. 3*C*-left panel). However, an unrelated lncRNA, NORAD, was not observed in the immunoprecipitate, suggesting the specificity of FIRRE-HuR interaction (Fig. 3*C*-right panel). Further, we did not detect FIRRE in the RIP assay performed with unrelated antibody DEAD-box helicase 24 (DDX24) or methyltransferase like 3 (METTL3) (Fig. 3*D*), verifying the specific interaction between FIRRE and HuR. Thus, our data strongly demonstrated a direct interaction between FIRRE and HuR.

### LncRNA FIRRE - HuR axis regulates cyclin D1 signaling

**Figure 4:**
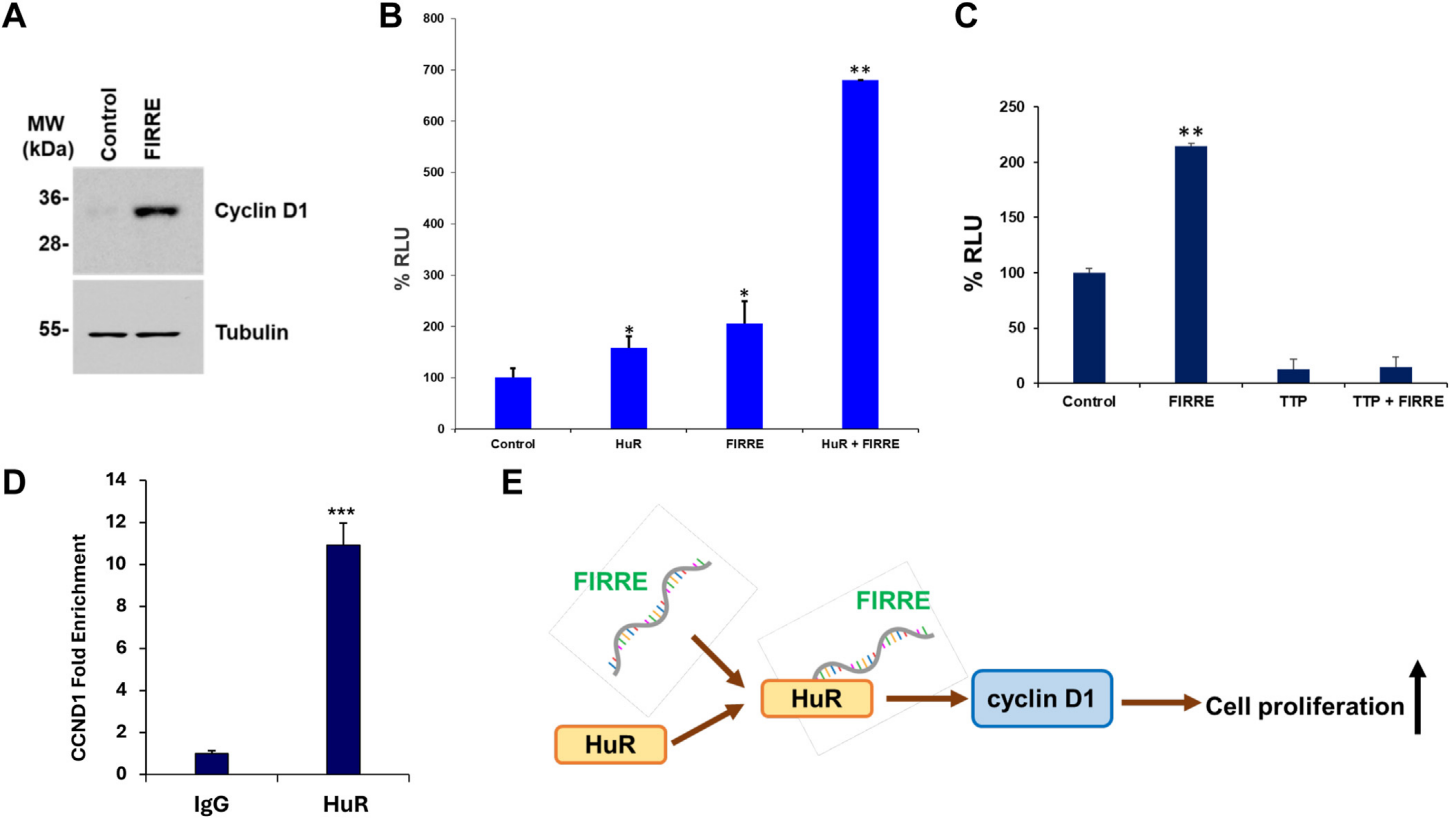
**FIRRE-HuR interaction regulates cyclin D1 signaling.***A*, control or FIRRE plasmid DNA overexpressing Huh7 cell lysates were subjected to Western blot analysis for cyclin D1 expression using a specific antibody. The membrane was reprobed with an antibody against tubulin as an internal control (n = 2). *B*, the 293T cells were cotransfected with 3′UTR of cyclin D1 fused with luciferase gene, HuR, FIRRE, or in combination. Luciferase activity was measured 24 h post-transfection. Experiments were repeated two times with three technical replicates. *C*, a similar experiment was performed with 3′UTR of cyclin D1 fused with luciferase gene, TTP, FIRRE, or in combination. Luciferase activity was performed 24 h post-transfection. Experiments were repeated two times with three technical replicates. *D*, RNA isolated from immunoprecipitates using isotype control (IgG) or HuR antibody in Huh7 cells was used to perform enrichment of cyclin D1 by qRT-PCR. n = 3 biological replicates and three technical replicates. Data information: Data were analyzed by Student's *t* test. Small bars indicate SD. (∗*p* < 0.05; ∗∗*p*< 0.01; ∗∗∗*p* < 0.001). *E*, a schematic diagram showing the mechanism of enhanced cell proliferation by lncRNA FIRRE (*arrow* denotes upregulation).

HuR is known to bind 3′UTR of ARE motifs in cyclin D1 mRNA and stabilize the protein (23). Since FIRRE overexpression promotes tumor growth, we examined the status of cyclin D1 expression for a mechanistic relationship. We observed that FIRRE overexpression increases cyclin D1 expression (Fig. 4*A*). We next examined whether FIRRE-HuR interaction enhances the stabilization of cyclin D1. For this, we used 3′UTR of cyclin D1 fused with the luciferase gene and cotransfected with HuR, FIRRE or in combination. Our results showed that HuR or FIRRE enhances the luciferase activity, and when both HuR and FIRRE were co-transfected, we observed enhanced luciferase activity suggesting an increased cyclin D1 stabilization (Fig. 4*B*). TTP degrades cyclin D1 by binding through the ARE motif of 3′UTR (24). To further verify our findings, we used TTP as a control and performed similar experiments. As expected, TTP overexpression reduces the luciferase activity, and co-expression with FIRRE did not rescue the TTP-mediated activity (Fig. 4*C*). Next, we verified the interaction between cyclin D1 and HuR using RIP with antibody against HuR, followed by qRT-PCR. IgG antibody was used as a negative control. Cyclin D1 RNA was significantly enriched in Huh7 immunoprecipitates with HuR antibody (Fig. 4*D*). HuR also stabilizes catenin mRNA and protein (25). We therefore examined the effect of FIRRE-mediated catenin activity with the TOPflash reporter assay, as described earlier (26). TOPflash and FOPflash activities were measured following FIRRE siRNA transfection (50 nM) in HepG2 cells for 48 h. We did not observe any changes (Fig. S4), suggesting that FIRRE may not be directly involved in -catenin activity. Together the findings provided the molecular insight that the FIRRE-HuR axis enhances cell proliferation by increasing cyclin D1 expression (Fig. 4*E*).

### Increased lncRNA FIRRE expression in HCC

**Figure 5:**
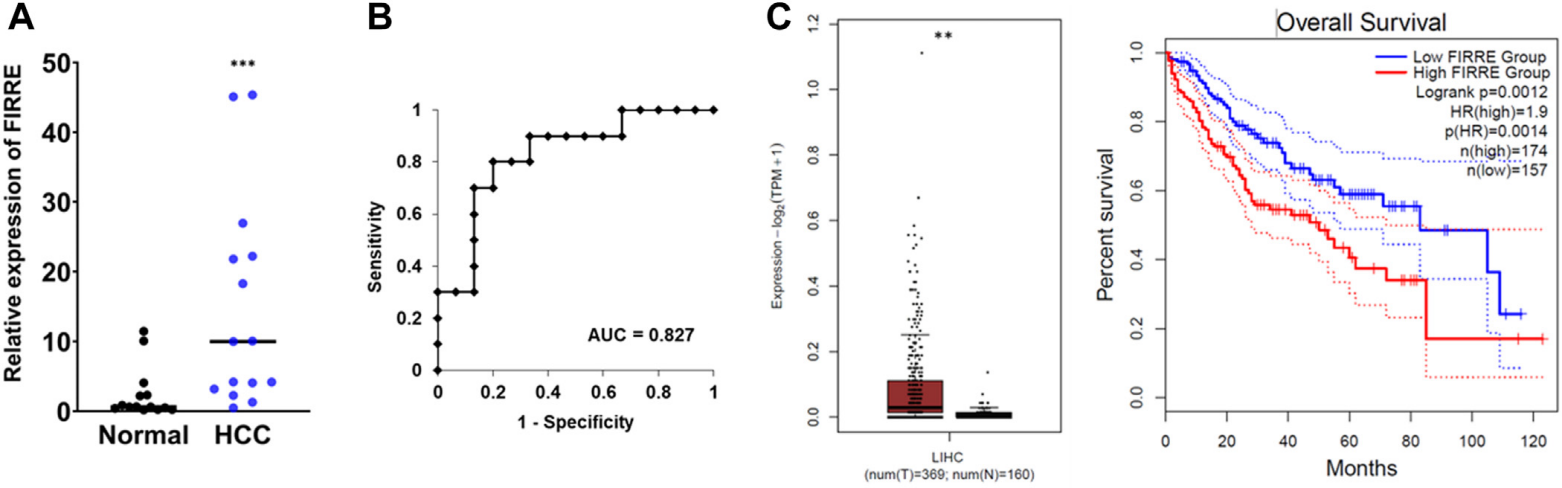
**FIRRE is highly expressed in HCC specimens.***A*, RNAs isolated from normal liver (n = 14), and HCC (n = 16) samples were used for qRT-PCR with three technical replicates. 18S rRNA was used as an internal control. Dot plots present relative expression of FIRRE of normal tissues *versus* HCC tissues. The *line* indicates the median value per group. Statistical analysis was performed using a two-tailed Student’s *t* test; (∗∗∗*p* < 0.0003). *B*, ROC curve with corresponding AUC for FIRRE is discriminating HCC from normal liver tissues. *C*, the relative expression level of FIRRE and survival plot in liver cancer from the TCGA database are shown.

We observed a higher expression of FIRRE in HCV-cured HCC. We next asked whether lncRNA FIRRE is higher in HCC irrespective of etiology. To test this, we examined the status of FIRRE in diverse resected HCC specimens and found that relative FIRRE expression was enhanced in the majority of the HCC specimens when compared to normal liver tissues (Fig. 5*A*). Normal liver samples were collected from donors with no known liver disease. FIRRE had an area under the curve (AUC) of 0.8267 ± 0.0874 (95% CI = 0.655–0.998) (Fig. 5*B*). The optimal cut-off value of dCT was indicated at 16.24, with a sensitivity of 80% and specificity of 80%. Further, the cancer genome atlas (TCGA) data analysis indicated that HCC patients with high FIRRE levels had significantly poorer overall survival than those with a low FIRRE level (Fig. 5*C*). Thus, our data indicated that FIRRE expression was higher in HCC irrespective of etiology.

### LncRNA FIRRE expression in HCC-PDX tissues and liver cancer tissue microarray

**Figure 6:**
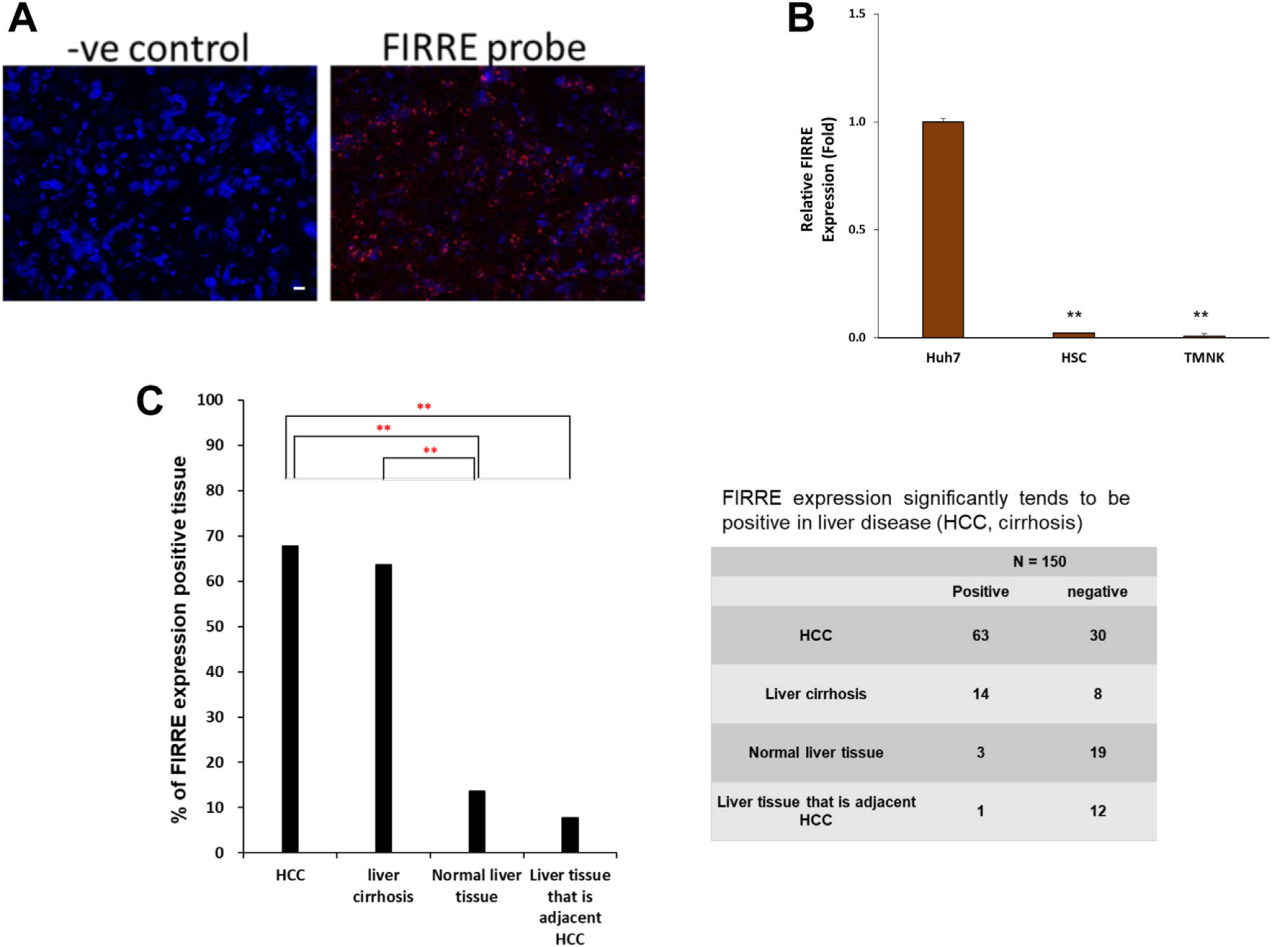
**FIRRE is expressed ubiquitously in the tumor mass.***A*, representative image of FISH analysis with an RNA probe of FIRRE (*red*) and DAPI staining (*blue*) of formalin-fixed paraffin-embedded P0 HCC-PDX tissue. An unrelated probe was used as a negative control (magnification: 40× and scale bar 10 μm). *B*, RNAs from indicated cells were used for FIRRE expression. Relative gene expression is shown with mean ± SD. n = 2 biological replicates and three technical replicates. *C*, liver cancer TMA of FIRRE. FISH analysis of TMA stained with FIRRE probe in human HCC TMA slides. The bar graph presents the percent of FIRRE expression positive tissue among each core. Significance was determined using the chi-square test (∗∗*p* < 0.01).

We next examined the localization of FIRRE in formalin-fixed HCC-PDX tissues obtained from our previous study (27). *In situ* hybridization using RNAScope suggested that FIRRE is localized ubiquitously in the tumor mass of P0 HCC-PDX tumor (Fig. 6*A*). Further analysis using expression of FIRRE in Huh7 cells, primary hepatic stellate cells (HSC), and immortalized sinusoidal TMNK-1 cells showed an elevated FIRRE expression only in cells of hepatocyte origin, and not in HSC or TMNK-1 cells (Fig. 6*B*). We subsequently examined the FIRRE expression in liver cancer TMAs that include samples from HCC, adjacent non-tumor tissues, and normal liver. *In situ* hybridization studies were performed in cut sections of two separate liver cancer TMAs (US BioMax Inc-BC03116a, and BC03117a) with FIRRE probe and RNAScope Multiplex Fluorescent Reagent. Patterns of FIRRE differential staining intensity were scanned by a digital imaging scanner and observed a differential expression (Fig. 6*C*). Higher FIRRE expression was displayed in 63/93 HCC and 14/22 cirrhotic tissue cores. Interestingly, 4/35 normal or non-tumor tissues adjacent to tumor tissue cores exhibited FIRRE expression. Corresponding hematoxylin and eosin-stained slides suggested that these tissues' core may not have normal histology. Taken together, these results suggested that lncRNA FIRRE promotes HCC progression, acts as an oncogene, and may have potential as a biomarker for HCC.

## Discussion

In this study, we have identified that the lncRNA FIRRE is highly expressed in HCV-associated HCC, even after clearing the virus by DAA treatment. Overexpression of FIRRE in hepatocytes induces cell proliferation and accelerates xenograft tumor growth. We showed that FIRRE interacts with RNA binding protein HuR and enhances HuR-cyclin D1 signaling. LncRNA FIRRE is enhanced in HCC tissues, irrespective of etiology. Our results demonstrated that lncRNA FIRRE has a potential role in HCC growth and may serve as a promising biomarker.

LncRNA FIRRE has been detected in several cancers. FIRRE is a relatively new identified non-coding RNA and its role in HCC is emerging. Overexpression of FIRRE promotes diffuse large B-cell lymphoma, and colorectal cancer (28, 29). FIRRE appears to be a tumor-promoting lncRNA, and future studies involving hepatocyte-specific overexpression of FIRRE in genetically modified mouse models may reveal novel phenotypic effects. The lncRNA FIRRE expression was elevated in hepatocellular carcinoma and associated with MBNL3 for promoting the splicing activity of PXN or PFKFB4 for glycolysis (30, 31). However, due to the diversity of functional consequences of lncRNAs, the specific mechanism of FIRRE in HCC remains to be further investigated. We observed cytoplasmic and nuclear localization of FIRRE in the HCC PDX xenograft tumor section. The FIRRE revealed potential for hepatocyte growth, colony-forming ability, and migration *in vitro*. We also found that Huh7 cells expressing FIRRE implanted into nude mice display faster tumor growth. However, in our experimental system, we did not observe the alteration of hexokinase or pyruvate kinase M2 in FIREE overexpressing HCC cells (30), most likely using different cell origins.

Recent studies have suggested that lncRNAs are active biological molecules and can drive carcinogenesis by promoting cell proliferation by regulating critical proteins, such as cyclins and c-Myc. HuR is a ubiquitously expressed RNA-binding protein in many types of cells and plays an important role in the regulation of mRNA stability. HuR binds to a specific single-stranded motif in ARE primarily located in the 3′ untranslated regions of early response genes (32). HuR is highly expressed in HCC and regulates cell proliferation and survival (33). Increased HuR expression is closely associated with unfavorable prognosis in various cancer types (34). We observed the expression of HuR in Huh7-FIRRE xenograft tissues by Western blot analysis (Fig. S5). HuR interacts with FIRRE and binds to the 3′UTR of cyclin D1. We show mechanistically that FIRRE interacts with RNA binding protein HuR and enhances HuR-cyclin D1 signaling (Fig. 4*E*). Cyclin D1 works as a controller of cellular growth and has functions for cancer progression. We also observed increased expression of VEGF and Oct4 in Huh7-FIRRE xenograft tumors suggesting FIRRE may enhance angiogenesis and/or EMT state, although further work is needed to understand the role of FIRRE in metastasis signaling.

LncRNA are functional transcripts that regulate cancer progression through diverse mechanisms and serve various biological functions (35). LncRNA FIRRE is enhanced in HCC tissues, irrespective of cancer etiology. Receiver operating characteristic curves and the AUC were used to assess the feasibility of using FIRRE as a diagnostic tool for the detection of HCC. An optimal cut-off value of dCT was indicated as 16.24 (AUC = 0.8267; SE = 0.0874; 95% CI: 0.655–0.998), suggesting FIRRE may help as a complementary diagnostic candidate for HCC. A recent study suggested that FIRRE was upregulated in paired HCC tissues and adjacent tissues (n = 8) (31), which is in agreement with our results. Analysis of FIRRE expression from HCC specimens and TMA data suggest that FIRRE expression is higher in advanced liver disease. Clinical information of 102 HCC patients including in TMAs was provided in Table S1. There are few lncRNAs that have been tested as a diagnostic or prognostic and predictive biomarker in several cancers (36). Together, these data suggested that lncFIRRE has potential as an HCC biomarker.

In summary, our results demonstrated that FIRRE facilitates tumor cell proliferation, and induces cell-cycle progression. Mechanistically, the FIRRE interacts with HuR resulting in HuR target gene Cyclin D1 mediated upregulation cell growth. These findings indicate that FIRRE is a critical molecule for tumor progression and may be an effective target for HCC therapy. LncRNA research in liver cancer is in its infancy. A growing number of studies have shown that abnormal expression of lncRNAs influences cancer development, including HCC (18, 37). LncRNA mechanisms are emerging in various diseases and may also function as a potential biomarker for other cancers (NCT04946968, NCT03000764, NCT02641847). Further research is critical in advancing our understanding of lncRNA in liver cancer and applies in clinical practice.

## Experimental procedures

### HCC specimens

We have obtained coded 16 HCC specimens (resected) through institutional review board (IRB) approval at Saint Louis University (IRB protocol #27805) and Liver Tissue and Cell Distribution Center (LTCDS -University of Minnesota - Twin Cities). The study was approved by the Ethics Committee of Saint Louis University, and written informed consent was obtained from all the patients. We procured 15 normal liver samples from The National Disease Research Interchange and LTCDS. Normal liver samples came from donors with no known liver disease or viral infection. All tissue samples were preserved at −80 °C until RNA extraction was performed.

### Ethical approval and informed consent

The study was approved by the Ethics Committee of Saint Louis University. We have approval from the Saint Louis University Internal Review Board (IRB protocol #27805) for the use of the patient clinical samples from repositories.

### RNA isolation and quantitative real-time PCR

Total RNA was isolated from liver specimens using TRIzol reagent (Invitrogen) and complementary DNA (cDNA) was generated by reverse transcription using random hexamers and a SuperScript III reverse transcriptase kit (Invitrogen). For quantification of gene expression, quantitative real-time PCR (qRT-PCR) was performed with a 7500 real-time PCR system using SYBR-green PCR master-mix (Thermo Fisher Scientific) and specific primers (Table S2). The 18S ribosomal RNA (18S rRNA) was used as an internal control. The relative gene expression was analyzed by the 2^−ΔΔCT^ formula (ΔΔCT = ΔCT of the sample − ΔCT of the untreated control).

### Cell lines

Human kidney epithelial 293T (ATCC Cat# CRL-3216), human hepatoblastoma HepG2 (ATCC Cat# HB-8065), and human hepatocellular carcinoma (HCC) Hep3B (ATCC Cat# HB-8064) cells were purchased from ATCC. HCC Huh7 cells were obtained from Charles Rice (Washington University). HCC cell lines (HLE, HLF, and ALEX) were obtained from Tatsuo Kanda (Nihon University School of Medicine). Immortalized human hepatocytes (IHH) were generated in our laboratory (38). Cells were maintained in Dulbecco’s modified Eagle’s medium (DMEM) supplemented with 10% fetal bovine serum (FBS) and 1% penicillin-streptomycin at 37 °C in a 5% CO_2_ atmosphere.

### Cell proliferation, colony formation, and wound-healing migration assays

We received pcDNA3.1-FIRRE clone from Dr Zhuo (39) and recloned in pcDNA3 vector (life technology). Huh7 cells were transfected with pcDNA3 or pcDNA3-FIRRE plasmid DNA and selected with G418 (Sigma, 800 μg/ml), pooled for the establishment of stable cell lines (Huh7-vector or Huh7-FIRRE). The vector control or FIRRE overexpressed Huh7 cells (Huh7-FIRRE) were seeded on a 35-mm dish at a density of 10^4^ cells/dish. Cells were harvested at different time points, and live cell numbers were counted by the Trypan blue exclusion method using the BioRad TC10 counter. The proliferation assay was repeated at least three times. For colony formation assay, control or Huh7-FIRRE cells (3 × 10^2^) were seeded. Two weeks later, colonies were stained with 0.1% crystal violet and counted. Control or siRNA to FIRRE (20 nM) transfected HepG2 cells were grown to confluency and scratched using a pipette tip. Wounds were made for each sample. The migration distance of cells was photographed and measured at 0 time and after 24 or 48 h.

### *In vivo* tumor growth assay

Huh7-vector control or Huh7-FIRRE cells (1 × 10^6^) containing 40% Matrigel were injected subcutaneously into the flank of BALB/c athymic nude mice (7–8 weeks old, n = 4/group). Nude mice (CRL #553, NCI strain) were purchased from Charles River Laboratories. Body weight was monitored, tumor size was measured using a slide caliper, and volume was calculated using the formula ½ *L* × *W*^2^. All the mice were sacrificed after 20 days of tumor cell injection. All animal experiments were conducted in accordance with the NIH guidelines, following a protocol (#2463) approved by the Institutional Animal Care and Use Committee of Saint Louis University.

### RNA pull-down and mass spectrometry

To identify the binding partner of FIRRE, pulldown and mass spectrometry analyses were performed as described previously (40). Briefly, FIRRE sense or antisense RNA was *in vitro* transcribed from pcDNA3 FIRRE plasmid DNA (500 ng) using biotin RNA labeling mix and T7 or SP6 RNA polymerase (AmpliScribe T7-Flash Transcription kit, Lucigen) following the manufacturer’s instruction. Purified biotinylated sense or antisense RNA (20 pmol) was labeled with streptavidin magnetic beads (ThermoFisher Scientific) for 1 h in the rotor at room temperature. Huh7 cells were lysed in immunoprecipitation (IP) buffer (25 mM Tris-HCL pH 7.5, 150 mM NaCl, 1 mM EDTA, 5% glycerol, 1% NP-40, 1× protease inhibitor, and 100 U/ml RNase inhibitor) and RNA pull-down assay was performed by Pierce Magnetic RNA-Protein Pull-Down Kit (ThermoFisher Scientific). Cell lysates (5 mg) were incubated with beads containing sense or antisense RNA for 3 h at 4 °C. After washing the beads, the bound proteins were eluted with elution buffer at 37 °C for 1 h with agitation. The eluted supernatant against sense/antisense strand was examined by LC-MS and Western blot analysis. The LC-MS was done in the proteomics core facility, at Washington University. The mass spectrometer used for data acquisition is a Thermo Q-Exactive system. Peptides were separated on an EASYnLC system with a Thermo ES803 PepMap C18 column; data were acquired in data-dependent acquisition mode (top 10 m/z for MS2 per cycle). Candidate proteins were defined as those that have at least 2-fold enrichment at the sense strand as compared with antisense RNA pull-down. In another set of experiments, the eluted supernatant along with total lysates were subjected to Western blot analysis.

### Western blot analysis

Control empty vector or FIRRE overexpressed cells were lysed in a sample buffer, subjected to SDS-PAGE, and transferred onto a nitrocellulose membrane. The membrane was blocked in 5% nonfat dried milk and incubated with a specific primary antibody overnight at 4 °C followed by incubation with a secondary antibody conjugated with horseradish peroxidase (HRP) for 1 h. Proteins were detected by an enhanced chemiluminescence Western blot substrate (ThermoFisher Scientific). Commercially available antibodies to cyclin D1 (Santa Cruz Biotechnology Cat# sc-8396, 1:1000) and HuR (Santa Cruz Biotechnology Cat# sc-5261, 1:1000), TTP (Sigma-Aldrich Cat# T5327, 1:1000), Actin- HRP antibody (Santa Cruz Biotechnology Cat# 517582, 1:5000), and tubulin (Santa Cruz Biotechnology Cat# sc-58666, 1:2000) were used for Western blot analysis.

### RNA immunoprecipitation (RIP)

Huh7 cells were transfected with vector or pcDNA3-FIRRE plasmid DNA and cells were lysed after 24 h of transfection with specific IP buffer (25 mM Tris-HCL pH-7.5, 150 mM NaCl, 1 mM EDTA, 5% glycerol, 1% NP-40, 1× protease inhibitor, and 100 U/ml RNase inhibitor). Cell lysates were centrifuged and incubated overnight with 1 μg of antibody against HuR (SantaCruz) or the isotype control antibody at 4 °C. Cell lysates were incubated with protein G Sepharose beads (Amersham Biosciences) for 2 h. After washing, RNA was isolated from beads using TRIzol reagent, cDNA was synthesized, and the relative expression of FIRRE and NORAD (41) was examined by qRT-PCR as described previously.

### Luciferase assay

293T cells were transfected with 3′UTR of cyclin D1 fused with luciferase gene, HuR, FIRRE, TTP, or respective controls for luciferase assay. Cell extract was prepared 24 h after transfection, and relative luciferase activity was determined as previously described (42).

### RNA *in-situ* hybridization

RNA *in-situ* fluorescence hybridization (FISH) was performed on the formalin-fixed paraffin-embedded (FFPE) tumor tissue (40) using the RNAScope Multiplex Fluorescent Kit v2, 323100 (Advanced Cell Diagnostics) with probes (Hs-FIRRE targeting 2–453 of NR_110426.1). FFPE tissue blocks were sectioned from P0 HCC patient-derived xenograft (PDX) (27) and deparaffinized with xylene. RNAScope hydrogen peroxide was added to the slide and incubated for 10 min at room temperature. After washing with PBS, tissues were permeabilized using protease for 10 min at room temperature, and hybridization to the RNA probe was performed by incubation in the oven for 2 h at 40 °C. For negative control, an unrelated RNA probe was used. After washing, the slides were processed for standard signal amplification steps as described previously (40). Slides were counter-stained with DAPI (4′,6′-diamidino-2-phenylindole) for nucleus staining, mounted, and observed under a fluorescence microscope.

### Tissue microarray for liver cancer

The expression level of FIRRE in liver cancer tissue microarrays (TMA) that includes samples from HCC, adjacent non-tumor tissues, and normal liver were examined. Two human liver cancer tissue microarrays (US Biomax: BC03116a and BC03117a), were purchased from US Biomax. Two slides contain 93 cores of liver cancer tissues, 22 cores of cirrhosis, 13 adjacent normal liver tissues, and 22 normal liver samples. *In situ* hybridization studies were performed in cut sections of HCC TMAs with FIRRE probe and employing RNAscope Multiplex Fluorescent Reagent. Patterns of differential staining intensity of FIRRE were scanned by a digital imaging scanner and analyzed.

### Statistical analysis

All the experiments were performed at least in duplicates or triplicates, and data are presented as mean ± standard deviation. Statistical analysis was performed by the Student *t* test with a two-tailed distribution. Statistical analysis for TMA data was performed using chi-square test. *p* value of <0.05 was considered statistically significant.

## Data availability

This study includes no data deposited in external repositories.

## Supporting information

This article contains supporting information.

## Conflict of interest

The authors declare that they have no conflicts of interest with the contents of this article.
