# Supplementary material for: Increased expression of long non-coding RNA FIRRE promotes hepatocellular carcinoma by HuR-CyclinD1 axis signaling
Source: J Biol Chem. 2024 Mar 29;300(5):107247. doi: 10.1016/j.jbc.2024.107247 (PMC11061211; doi:10.1016/j.jbc.2024.107247)
Supplement: Supplemental Figures and Tables [file mmc1.docx]

**Supporting Document**

**Increased expression of long non-coding RNA FIRRE promotes hepatocellular carcinoma by HuR-CyclinD1 axis signaling**

Yuki Haga^1^, Debojyoty Bandyopadhyay^2^, Mousumi Khatun^2#^, Ellen Tran^2^, Robert Steele^2^, Sumona Banerjee^2^, Ranjit Ray^1^, Mustafa Nazzal^3^, and Ratna B. Ray^2^

Departments of ^1^Internal Medicine, ^2^Pathology, and ^3^Surgery,

Saint Louis University, Missouri 63104, USA

| **HCC** | **N=102** |
| --- | --- |
| **Age** | **52.5 ± 10.7** |
| **Sex: Female (%)** | **23 (22.5)** |
| **Etiology: HCV / HBV / steatosis / unknown (%)** | **14 (13.7) / 4 (3.9) / 5 (4.9) / 6 (5.9) / 73 (71.6)** |
| **Stage: I / II / IIIA / IIIC / IVA / unknown (%)** | **4 (3.9) / 28 (27.5) / 48 (47.1) / 3 (2.9) / 5 (4.9) / 14 (13.7)** |
| **Grade: 1 / 2 / 3 / unknown (%)** | **14 (13.7) / 50 (49.0) / 17 (16.7) / 21 (20.6)** |

**Table S1. Clinical information of HCC patients including in TMAs**

**Table S2. Primers used in this study**

| Gene | Forward primer (5’-3’) | Reverse primer (5’-3’) |
| --- | --- | --- |
| FIRRE | CTGTGACCTCGCTTCACTTCT | GTGGCAAAGAGCAGAAGATAG |
| NORAD | AGCGAAGTCCCGAACGACGA | TGGGCATTTCCAACGGGCCAA |
| VEGF | CGAAACCATGAACTTTCTGC | CCTCAGTGGGCACACACTCC |
| Oct4 | TCGAGAACCGAGTGAGAGG | GAACCACACTCGGACCACA |
| 18S rRNA | GTCATAAGCTTGCGTTGATT | TAGTCAAGTTCGACCGTCTT |


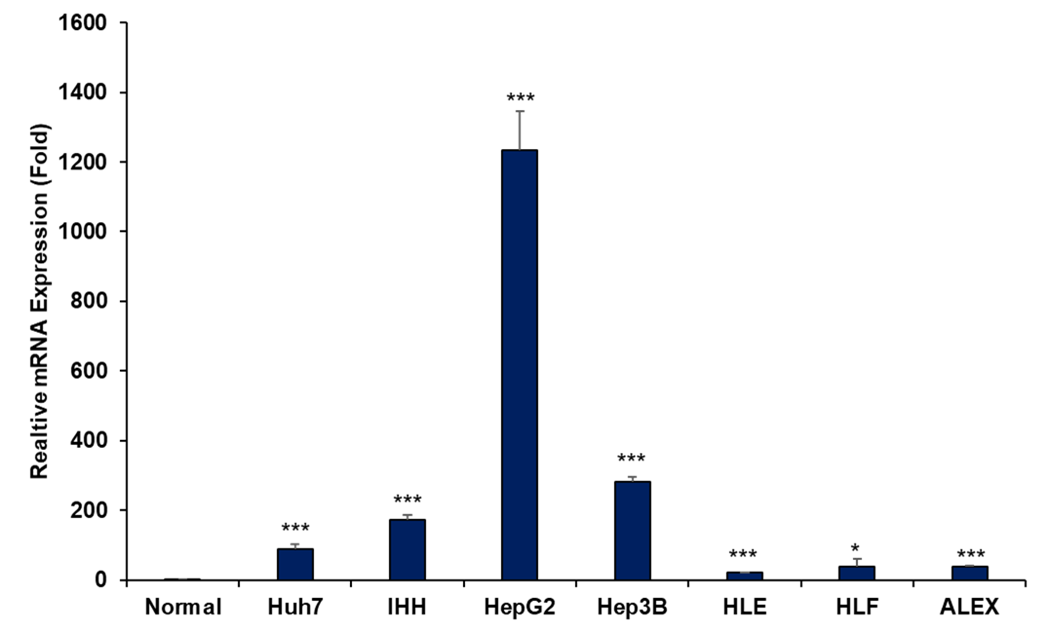


**Figure S1:** Relative expression of FIRRE in HCC cell lines analyzed by qRT–PCR. 18S rRNA was used as an internal control. n = 2 biological replicates and three technical replicates. Data information: Data were analyzed by Student’s t-test. Small bars indicate SD. (*p < 0.05; ***p < 0.001).


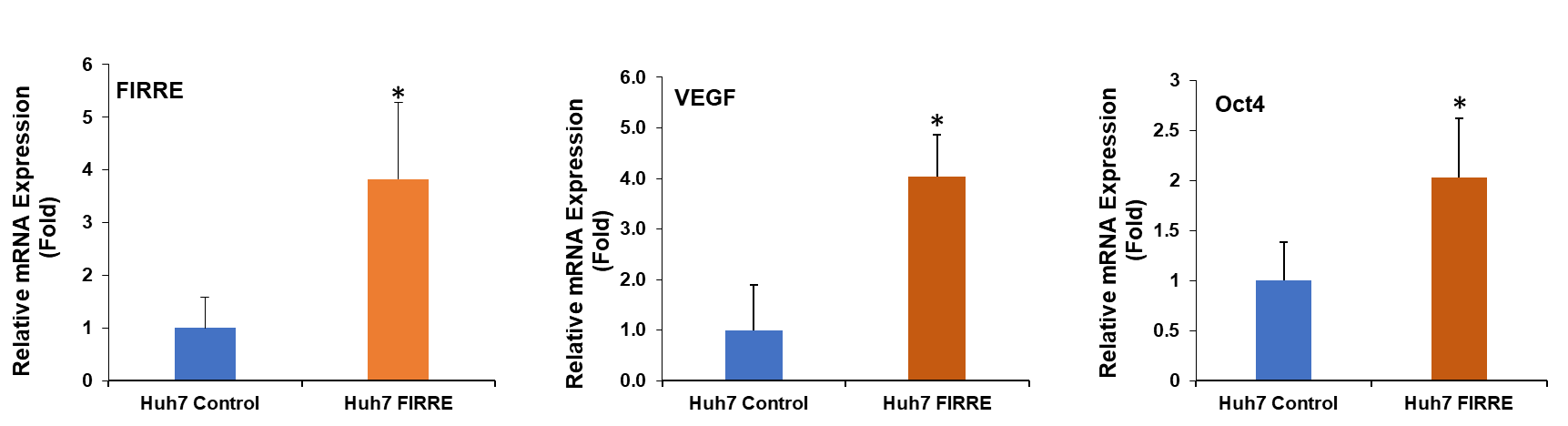


**Figure S2:** FIRRE, VEGF, and Oct4 expression were examined by qRT-PCR from RNA of Huh7-FIRRE xenograft tumors compared to Huh7 control tumors. 18S gene was used as an internal control. n = 4 animals per group and two technical replicates presented as the mean ± SD. Data information: Data were analyzed by Student’s t-test. (*p < 0.05).


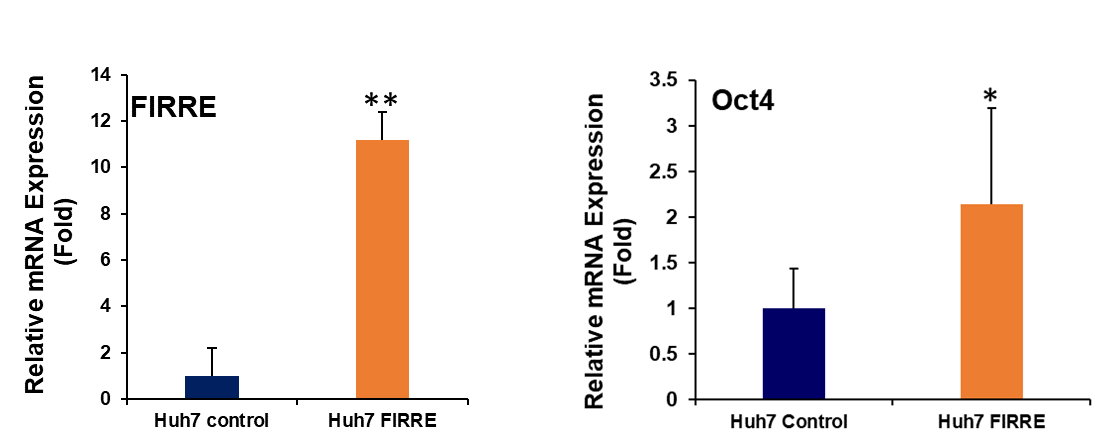


**Figure S3:** FIRRE and Oct4 expression were examined by qRT-PCR from RNA of control or Huh7-FIRRE stable. 18S gene was used as an internal control. Three technical replicates are presented as the mean ± SD. Data were analyzed by Student’s t-test. (*p < 0.05).


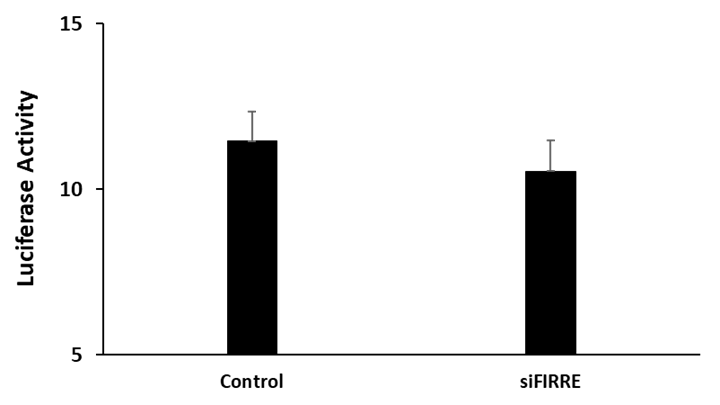


**Figure S4:** TOPflash reporter assay demonstrates no detectable changes in beta-catenin transcriptional activity in HepG2 cells following FIRRE siRNA transfection. The data are presented as TOPflash/POPflash ratio from triplicate experiments.


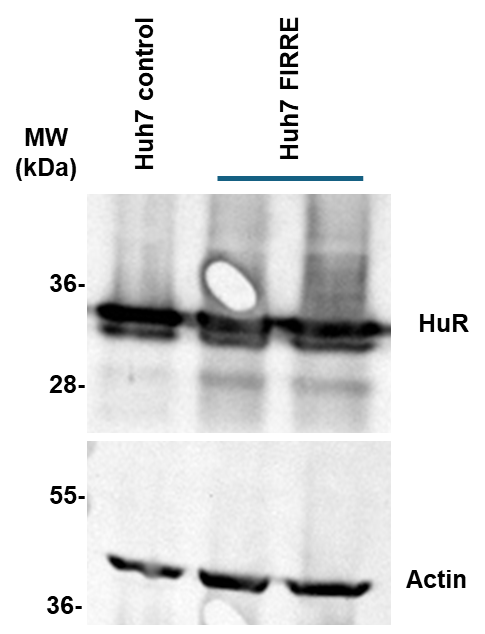


**Figure S5:** Western blot analysis for HuR using specific antibody in indicated samples. The blot was reprobed with actin antibody for protein load.
